# Elevated Expression of Inhibitor of Apoptosis-stimulating Protein of p53 (iASPP) and Methyltransferase-like 3 (METTL3) Correlate with Poor Prognosis in FIGO Ib1-IIa Squamous Cell Cervical Cancer

**DOI:** 10.7150/jca.41029

**Published:** 2020-02-10

**Authors:** Fang Wu, Yong Zhang, Yeying Fang, Shanshan Ma, Hua Zheng, Kang Liu, Rensheng Wang

**Affiliations:** 1 Department of Radiation Oncology, First Affiliated Hospital of Guangxi Medical University, Nanning 530021, Guangxi, China; 2 Life Science Institute, Guangxi Medical University, Nanning 530021, Guangxi, China; 3 Graduate School, Guangxi Medical University, Nanning 530021, Guangxi, China

**Keywords:** Cervical neoplasms, iASPP protein, human, METTL3, protein, human, prognosis, survival analyses

## Abstract

**Background**: Clinical outcomes of patients with early stage cervical cancer are determined by unique molecular events. Therefore, exploring novel biomarkers for the diagnosis and prognosis of cervical cancer is essential for guidance of cervical cancer treatment.

**Methods**: Patients with FIGO Ib1-IIa cervical cancer who were treated with radical hysterectomy at the first affiliated hospital of Guangxi Medical University, China were included in the study. iASPP and METTL3 expression in the tumor specimens and adjacent non-tumor cervical tissues was determined by immunohistochemistry and western blot, and its relationship with clinicopathologic factors and prognosis of cervical cancer patients was analyzed.

**Results**: Of 112 patients, 41 were stage Ib1, 22 Ib2, and 49 IIa. Their mean age was 45.3 years (range 25-75 years). Tumor size was 0.3 to 5.0 cm (mean 2.8 cm). Mean follow-up was 56.6 months (range 19-72 months). iASPP and METTL3 were higher in cervical cancer than normal cervix samples (p<0.001 and p<0.01, respectively). iASPP and METTL3 overexpression correlated with higher FIGO staging (p=0.013 and p=0.039, respectively), pelvic lymph node metastasis (p=0.002 and p=0.001, respectively), and poor 5-year recurrence-free survival, distant metastasis-free survival, progression-free survival, and overall survival rates (p=0.002, p=0.007, p=0.001, p=0.016, p=0.001, p<0.001, p=0.037, and p=0.042, respectively). High iASPP and METTL3 expression were independent prognostic factors (all p<0.05). The expression of iASPP was positively related with METTL3 (p=0.002).

**Conclusions**: iASPP and METTL3 levels were elevated in cervical cancer, and they were both independent indicators for poor prognosis in early stage cervical cancer patients.

## Introduction

Cervical cancer is the fourth most frequently diagnosed cancer and the fourth leading cause of cancer-related death in the world 1. Although the global incidence of cervical cancer is declining due to the widespread use of Pap smear screening tests, it remains one of the leading cancer-related causes of death for women 1. Therefore, exploring novel biomarkers for the diagnosis and prognosis of cervical cancer is essential for guidance of cervical cancer treatment.

Inhibitor of apoptosis-stimulating protein of p53 (iASPP) was initially identified as Rel-associated inhibitor (RAI), as its interaction with nuclear factor kappa B (NF-κB) subunit p65 (RelA) suppresses its transcriptional activity 2. iASPP acts as a key promoter of cell proliferation, epithelial-mesenchymal transition, invasion and cancer stemness by interacting with p53 to suppress p53-mediated transcription of target genes 3, 4. iASPP is overexpressed in many human cancers, including cervical cancer, leukemia, ovarian clear cell carcinoma, hepatocellular carcinoma, and non-small cell lung cancer 5-9. The increased expression of iASPP confers proliferative, migratory, and invasive capacities to cancer cells, and is associated with advanced stage, lymph node metastasis, chemoresistance, radioresistance and decreased survival 10.

Methyltransferase-like 3 (METTL3), a major RNA N6-adenosine methyltransferase, participates in the tumorigenesis of various tumors. Transcriptome-wide m6A profiling further showed that m6A modification is presented in thousands of RNA transcripts with unique distribution patterns 11, 12. However, the functional roles of m6A methylation in cancer initiation and progression remain to be determined. METTL3 is significantly increased in gastric cancer tissues compared with control in big crowd data sets and served as a poor prognostic factor for patients with gastric cancer 13. The expression of METTL3 gradually increased with the progress of tumor stage and grade, but METTL3 knockdown inhibited cell proliferation, migration and invasion in human gastric cancer cells 14. Moreover, knockdown of METTL3 also inhibited the growth of lung cancer by inducing apoptosis via PI3K/AKT pathway 15. These data suggest that METTL3 plays an important role in the progression of cancer and may act by inhibiting apoptosis. However, the expression of METTL3 and the relationship between METTL3 expression and clinicopathological characteristics or prognosis of cervical cancer have not been shown.

Prior studies suggest iASPP and METTL3 have a key role of tumorigenesis and prognosis of cancer. Both iASPP and METTL3 appear to be related to apoptosis and there may be a correlation between the two proteins. We hypothesized that the combination of iASPP and METTL3 would promote growth, invasion and migration via inhibiting apoptosis of cancer cells. However, the correlation between iASPP and METTL3 is not clearly defined in cervical cancer. In this study, we investigated the clinical significance of iASPP and METTL3 expression and association between the two proteins in cervical cancer.

## Materials and Methods

### Patients

A total of 112 patients with cervical cancer were enrolled from patients treated at the first affiliated hospital of Guangxi Medical University in China between January 2013 and December 2015. The criteria for patient inclusion were as follows: (1) pathologically confirmed patients with FIGO Ib1-IIa (2009 FIGO staging) squamous cell cervical cancer; (2) radical hysterectomy and bilateral pelvic lymph-node (LN) dissection were performed in our hospital with or without preoperative adjuvant therapy, patients who had matched fresh surgical specimens and adjacent normal cervical tissues; (3) no previous history of other cancers; (4) the clinical data and the follow-up information were complete. The exclusion criteria included: (1) patients who underwent palliative surgery; (2) patients who had distant metastasis or peritoneal dissemination that was confirmed during the operation. Prior informed consent was obtained from all patients, and the study was approved by the Ethics Committee of The First Affiliated Hospital of Guangxi Medical University.

### Study design

This was a retrospective analysis of the clinical data of patients with cervical cancer. The expression levels of iASPP and METTL3 were analyzed in the surgical tumor specimens and adjacent non-tumor cervical tissues specimens and then the patients were grouped according to their level of expression of the two proteins.

### Treatment regimens

Among the patients, 35 (31.3%) cases received 2-3 cycles of chemotherapy before operation. The chemotherapy regimens were paclitaxel at 175 mg/m^2^ on day 1, cisplatin at 60 mg/m^2^ on day 1; repeated every 3 weeks. Concurrent chemoradiation therapy was added after the operation in cases with risk factors such as pelvic LN metastasis, positive resection margin and parametrial invasion. The chemotherapy regimen was cisplatin 40mg/m^2^ on day 1 per week with a total of 4-5 cycles. The total dose was 50 Gy delivered at 2.0 Gy per daily fraction, with five fractions per week.

### Data collection

Clinicopathological data including age, FIGO staging, tumor size, lymphovascular space invasion (LVSI), deep cervical stromal invasion, pelvic lymph node metastasis and the follow-up time and results were collected. The final follow-up was performed in January 2019.

### Definitions

The recurrence-free survival (RFS) was defined as the time from treatment until any recurrence, either local or regional. Distant metastasis-free survival (DMFS) was defined as time from treatment until any distant metastasis due to any cause. Progression-free survival (PFS) was defined as the time elapsed between treatment initiation and local or regional progression or death or metastatic tumor progression from any cause. Overall survival (OS) was defined as time from treatment until death by any cause. Total follow-up time was defined as time in months from date of operation to the last clinic visit.

### Immunohistochemistry

All paraffin embedded sections were cut at 5-μm thickness followed by deparaffinization. Antigen recovery was performed in heat activated antigen retrieval pH 6 for iASPP and METTL3, and then specimens were incubated with 3% H_2_O_2_ for 10 min. Non-specific binding was blocked with protein block for 20 min at room temperature. The sections were incubated with rabbit polyclonal anti-iASPP antibodies (bs-0284R, 1:400, Bioss, Beijing, China) or with rabbit polyclonal anti-METTL3 antibodies (bs-17609R, 1:400, bioss, Beijing, China) at 4°C overnight, and then the sections were incubated with an anti-rabbit secondary antibody (Zhongshan Goldenbridge Biotechnology, Jiangsu, China) at 37°C for 30 min, washed with PBS for 5 min, and stained with diaminobenzidine for 5 min. Immunohistochemical staining was scored by using a four-point scale according to the percentage of positive cells: 0, <10% positive; 1+, 11%-25% positive; 2+, 26%-50% positive; 3+, >51% positive. The protein expression of iASPP and METTL3 in cervical cancer specimens was thus divided into a low-expression group (0 or 1+) and a high-expression group (2+ or 3+). Immunohistochemical analysis and scoring were performed by two independent investigators.

### Western blot

Total protein was extracted and separated by sodium dodecyl sulfate polyacrylamide gel electrophoresis and then transferred onto polyvinylidene difluoride membranes, which were blocked with 5% nonfat milk for 1-2 h at room temperature. The blotted membranes were incubated over night at 4°C with the following primary antibodies: rabbit anti-iASPP antibody (bs-0284R, 1:1000, Bioss, Beijing, China), rabbit anti-METTL3 (bs-17609R, 1:1000, Bioss, Beijing, China), and then was followed by incubation with anti-rabbit (1:5000) secondary antibody for 2h at room temperature. Bands were detected by enhanced chemiluminescence and exposure to X-ray films (FluorChem HD2, Santa Clara, CA USA).

### Statistical analysis

The statistical results were analyzed by using SPSS version 17.0 (SPSS Inc., Chicago, IL, USA). Numerical variables were recorded as means ± standard deviation (SD) and analyzed by independent t-tests. Categorical variables were presented as rates and analyzed by using the chi-square test or Fisher's exact test. The chi-square test was used to evaluate the association between iASPP and METTL3. Kaplan-Meier survival analysis was performed to determine the association of iASPP and METTL3 expression with survival, and the survival curves were compared between groups using log-rank tests. Multivariate Cox-regression analyses were conducted to identify independent prognostic factors. p<0.05 (two sides) were considered statistically significant.

## Results

### Baseline characteristics

Of the 112 patients with cervical cancer, 41 were classed as stage Ib1, 22 as Ib2, and 49 as IIa. The mean age of the patients was 45.3 years (range 25-75 years). The tumor sizes ranged from 0.3 to 5.0 cm (mean 2.8 cm). The mean follow-up time of surviving patients was 56.6 months (range 19-72 months).

### iASPP and METTL3 protein expression

**Figure 1:**
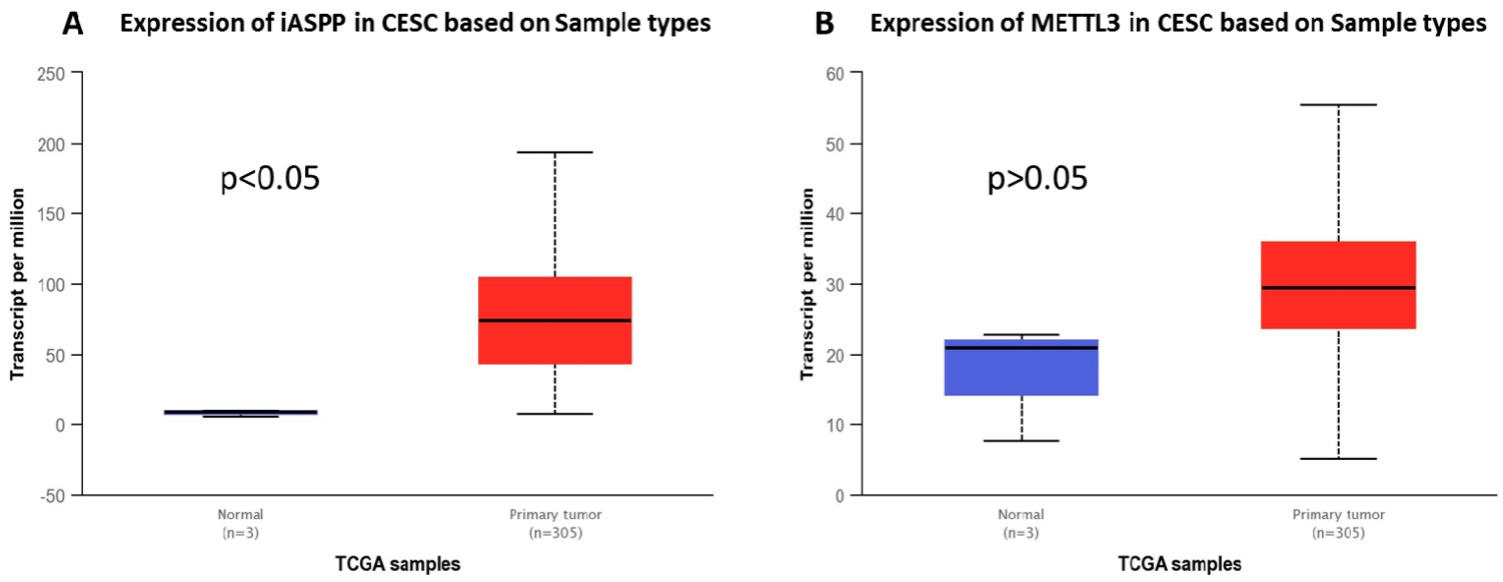
** iASPP and METTL3 expression in cervical cancer tissues (UALCAN data).** iASPP (A) and METTL3 (B) expression in cervical cancer tissues (UALCAN data, Red box for tumor tissue, n=305; blue box for normal tissue, n=3).

**Figure 2:**
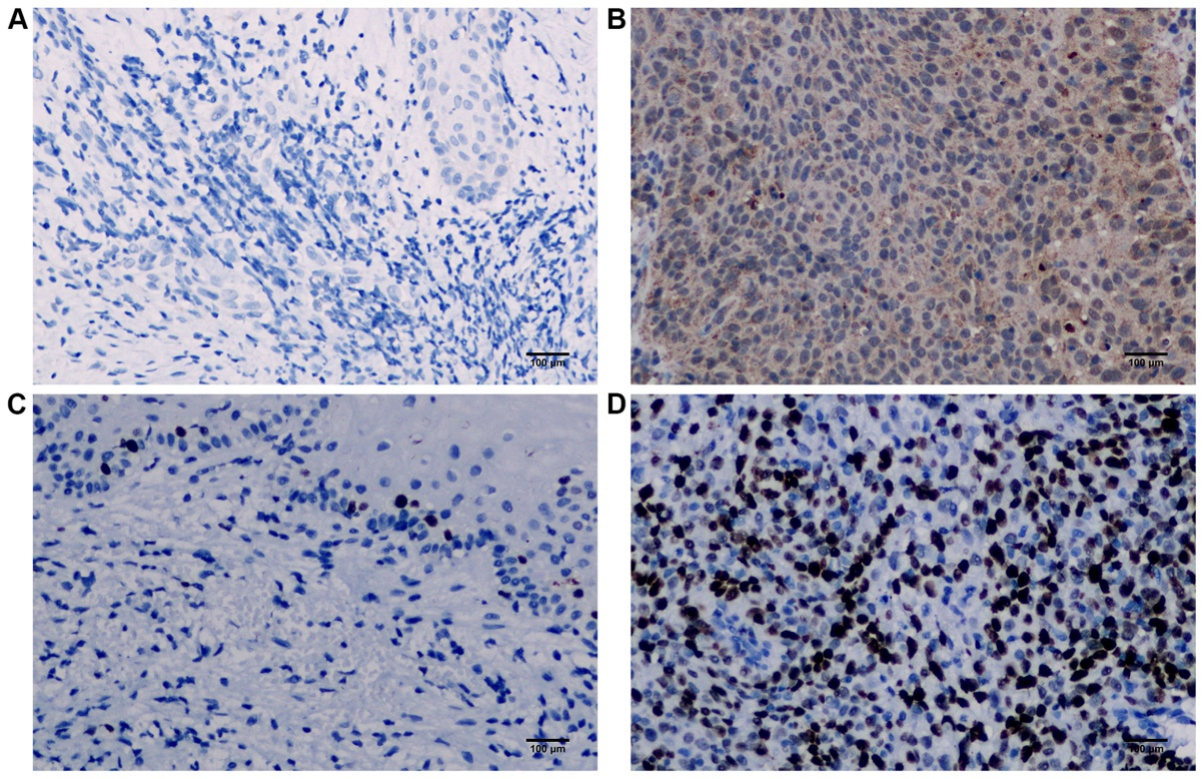
** iASPP and METTL3 expression in cervical cancer tissues.** Representative immunohistochemical images of iASPP negative in adjacent non-tumor cervical tissues (ANCT) (A) and positive in tumor tissues (B), METTL3 negative in ANCT (C) and positive in tumor tissues (D).

**Figure 3:**
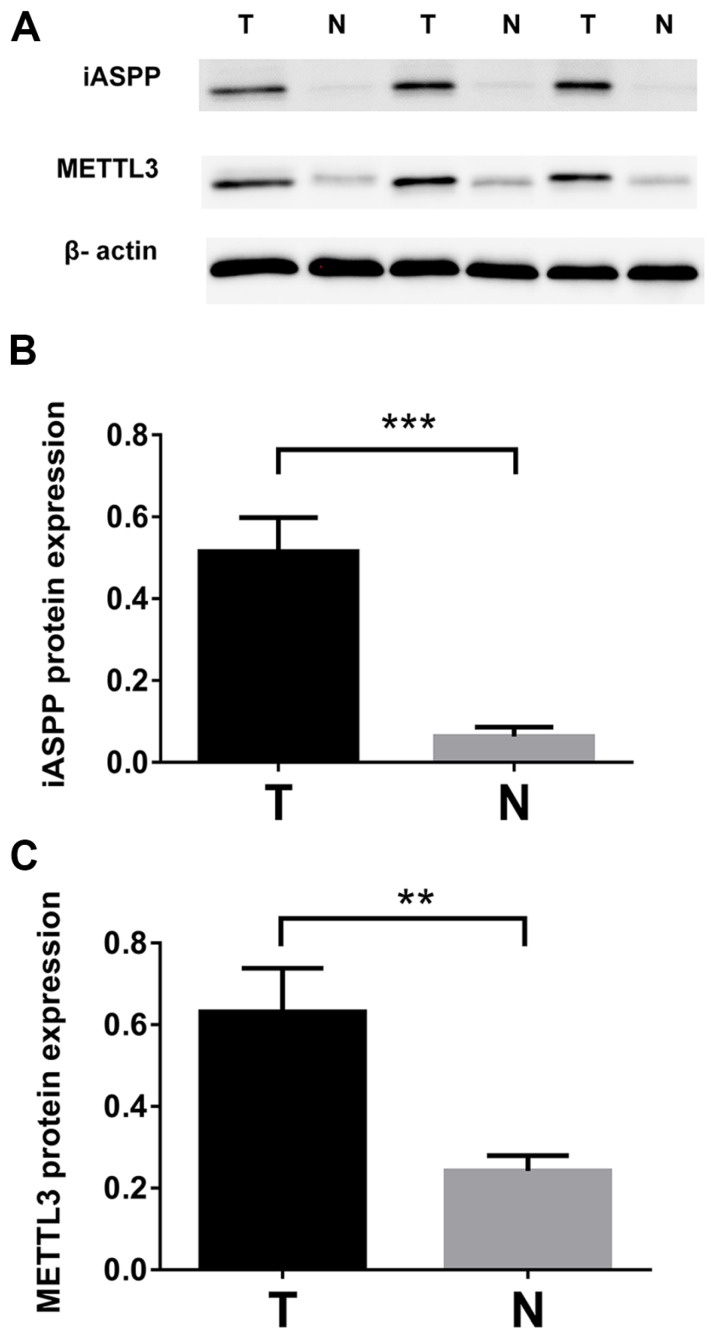
** Protein expression levels of iASPP and METTL3 in cervical cancer tissues and adjacent non-tumor cervical tissues (ANCT).** (A). iASPP and METTL3 protein expression levels were detected by western blot. The relative levels of iASPP. (B) and METTL3. (C) protein expression. (***p<0.001, **p<0.01).

We checked the expression of iASPP and METTL3 in the UALCAN datasets (http://ualcan.path.uab.edu/index.html), the data of which comes from TCGA, and found that iASPP expression is significantly elevated in cervical cancer compared with the normal tissues (Figure 1A). The expression of METTL3 was up-regulated in cervical cancer although no statistical significance was achieved (Figure 1B). We then examined the protein level of iASPP and METTL3 in the samples from a cohort of 112 cervical cancer patients by immunohistochemistry. In line with the UALCAN data, iASPP expression was significantly higher in tumor tissues than in adjacent normal cervical tissues (ANCT) (Figure 2A-B). Besides, METTL3 expression was dramatically elevated compared with normal tissues (Figure 2C-D). Consistent with the immunohistochemistry, iASPP and METTL3 protein expression in cancer tissues when quantified through western blot analysis in comparison with the β-actin loading control was also significantly higher than that in ANCT (Figure 3).

### Relationship between iASPP and METTL3 expression and clinicopathologic factors of cervical cancer

Of the cancer specimens, 69 of 112 cancers (61.6%) had high expression of iASPP and 65 of 112 cancers (58.0%) had high expression of METTL3. To investigate the association of iASPP and METTL3 expression with clinicopathologic characteristics we grouped the patients into high and low expression level groups and compared the differences between groups. The results indicated that the high iASPP and METTL3 expression was correlated with higher FIGO staging (p=0.013 and p=0.039, respectively) and pelvic lymph node metastasis (p=0.002 and p=0.001, respectively). There were no other correlations between iASPP and METTL3 expression and clinicopathologic characteristics (Table 1).

We next examined the association between iASPP and METTL3 expression. In 112 cervical cancers, iASPP expression presented a significant correlation with METTL3 (p = 0.002; Table 2).

### Prognostic significance of iASPP and METTL3 expression

**Figure 4:**
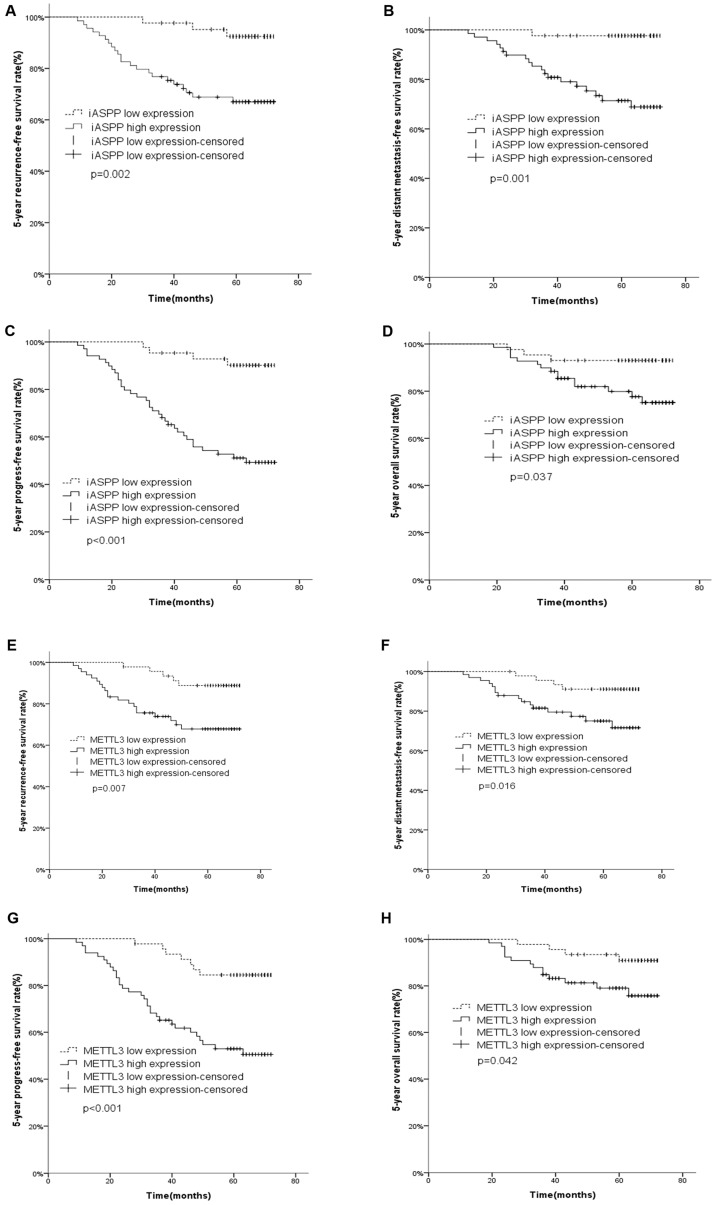
** Kaplan-Meier survival curves of iASPP and METTL3 expression in cervical cancer.** Cervical cancer patients with high iASPP expression had shorter 5-year recurrence-free survival (RFS) (A, p= 0.002), distant metastasis-free survival (DMFS) (B, p=0.001), progression free survival (PFS) (C, p<0.001) and overall survival (OS) (D, p=0.037) than those with low expression. Patients with high METTL3 expression had worse 5-year RFS (E, p= 0.007), DMFS (F, p=0.016), PFS (G, p<0.001) and OS (H, p=0.042) than those with low expression

The 5-year recurrence-free survival (RFS), distant metastasis-free survival (DMFS), progression-free survival (PFS) and overall survival (OS) rates were 67.0%, 68.1%, 49.3% and 75.2% in high iASPP expression and 92.5, 97.7%, 90.0% and 93.0% in low iASPP expression (p=0.002, <0.001, <0.001 and =0.037, respectively, Figure 4A-D).

In survival analysis with METTL3 expression, 20 recurrences, 16 distant metastasis, and 14 deaths occurred in 65 cases of iASPP high expression, while 5 recurrences, 4 distant metastasis and 4 deaths were observed in 47 cases of low expression. The 5-year RFS, DMFS, PFS and OS rates were 67.8%, 71.6%, 50.5% and 75.8% in high METTL3 expression and 88.8%, 91.1%, 84.5% and 91.0% in low METTL3 expression (p=0.007, =0.016, <0.001 and =0.042, respectively, Figure 4E-H).

Multivariate Cox analysis confirmed that high iASPP and METTL3 expression were independent poor prognostic factors for 5-year RFS (p=0.034 and 0.045, respectively), DMFS (p=0.005 and 0.001, respectively), PFS (p=0.004 and 0.001, respectively) and OS (p=0.006 and 0.004, respectively) in patients with early stage cervical cancer (Table 3). In addition, FIGO staging and pelvic lymph node metastasis were also identified as independent prognostic factors for 5-year RFS (p=0.001 and 0.008, respectively), DMFS (p=0.036 and 0.028, respectively), PFS (p<0.001 and p=0.017, respectively) and OS (p=0.020 and 0.040, respectively) (Table 3).

## Discussion

The results showed that both iASPP and METTL3 expression levels were higher in cervical cancer than normal cervix samples. iASPP and METTL3 overexpression correlated with higher FIGO staging, pelvic lymph node metastasis, and poor 5-year RFS, DMFS, PFS and OS rates when compared to those patients with low-expression. Multivariate Cox analysis indicated that high iASPP and METTL3 expression were both independent prognostic factors. The expression of iASPP was also positively related with METTL3.

In the present study, we detected iASPP expression profiles in early stage squamous cell cervical cancer and then analyzed the correlation of its expression with clinicopathologic factors and the long-term prognosis. Our results showed that elevated iASPP expression correlated with higher FIGO staging and more pelvic lymph node metastasis, and poor RFS, DMFS, PFS and OS. These data, consistent with previous studies 3-10, indicated that iASPP, as a newly identified oncoprotein, was involved in the carcinogenesis and progression of early stage cervical cancer and might serve as novel potential therapeutic target. Dong et al. 4 demonstrated that miR-124, directly targeting iASPP, reduces expression of iASPP and attenuated cervical cancer cell growth and invasiveness.

In this study we found that MRTTL3 was overexpressed in cervical cancer. The elevated expression correlated with higher FIGO staging and more pelvic lymph node metastasis, and poor RFS, DMFS, PFS and OS. METTL3 expression was also an independent prognostic factor. We presume that METTL3 is involved in a similar mechanism to other cancer types. Lin et al. 16 revealed that METTL3 enhances the translation of oncogenes and promotes the proliferation and invasion of lung cancer cells. Chen et al. 17 reported that METTL3 was significantly upregulated in human hepatocellular carcinoma (HCC) and was associated with poor prognosis. Knockdown of METTL3 drastically reduced HCC cell proliferation, migration, and colony formation in vitro. Knockout of METTL3 significantly suppressed HCC tumorigenicity and lung metastasis in vivo. These data suggested that METTL3 may act as an oncogene and participate in tumor progression.

Protein-protein interactions (PPI) are at the core of numerous cell death pathways such as apoptosis, autophagy, and necrosis 18. Affecting specific PPI involved in these pathways can change the fate of cells. iASPP plays an important role in regulating p53 dependent apoptosis and acts as a negative regulator of the tumor suppressor p53 19. iASPP overexpression was discovered in tumor tissues and moreover associated to poor prognosis and survival in some types of cancers 5-9. Targeted suppression of iASPP may serve as the mechanism by which its upstream protein prevents oncogenesis of cancer 19, 20. METTL3 was originally known to be responsible for m6A modification of mRNA. Recent studies have found that METTL3 plays important roles in a variety of tumors by regulating the translation of oncogenes 16, 21. METTL3 knockdown decreased Bcl2 and increased Bax and active Caspase-3 in gastric cancer cells, which suggested the apoptotic pathway was activated 14. The PI3K/Akt pathway is implicated in cell growth and survival, and Wei et al. 15 observed that knockdown of METTL3 changed the expression and phosphorylation of proteins of PI3K signaling pathway members. Besides, the Bax/Bcl-2 ratio in lung cancer cells was increased by the transfection of miRNA of METTL3, which suggested that apoptosis was inhibited in cancer cells. Based on these data, we raised the hypothesis that the interaction between iASPP and METTL3 is required for apoptosis activation in cancer cells. In this study, we preliminarily found that iASPP expression presented a significant correlation with METTL3. We speculate that this interaction may uncover a new layer in the highly complex regulation of cell death in cancer cells and open new avenues of exploration into the development of novel anticancer drugs that reactivate apoptosis in malignant tumors. However, this needs further study to confirm a direct interaction between the two proteins.

This study has some limitations. One limitation of this study is that some of the patients included in this study had neoadjuvant treatment, which might have effect on iASPP expression and outcome. This is only a preliminary finding on the correlation of iASPP and METTL3. The interaction between the two proteins is still unclear, and further study needs to be conducted to fully understand the potential involved molecular mechanism.

In conclusion, both iASPP and METTL3 protein expression levels were elevated in cervical cancer. iASPP and METTL3 are independent prognostic factors for poor survival in early stage squamous cell cervical cancer patients, suggesting that iASPP and METTL3 might serve as novel potential prognostic markers and therapeutic targets for treatment of cervical cancer. Moreover, iASPP expression presents a significant correlation with METTL3, which needs further study to fully understand the molecular mechanism involved.

## Figures and Tables

**Table 1:** Clinical/pathological characteristics of 112 cervical cancer patients grouped according to iASPP and METTL3 protein expression level.

Variables	Number of cases	iASPP expression level		P value	METTL3 expression level		P value
		Low (n=43)	High (n=69)		Low (n=47)	High (n=65)	
Age (years)				0.302			0.926
≤45	59	20	39		25	34	
>45	53	23	30		22	31	
FIGO staging				0.013			0.039
Ib1	41	23	18		22	19	
Ib2	22	7	15		11	11	
IIa	49	13	36		14	35	
Tumor size				0.269			0.106
≤4cm	79	34	48		37	42	
>4cm	33	9	21		10	23	
Deep cervical stromal invasion				0.061			0.409
No	45	22	23		21	24	
Yes	67	21	46		26	41	
Lymphovascular space invasion				0.231			0.097
No	47	15	32		24	23	
Yes	65	28	37		23	42	
Pelvic lymph node metastasis				0.002			0.001
No	63	32	31		35	28	
Yes	49	11	38		12	37	
Neoadjuvant chemotherapy				0.307			0.267
No	77	32	45		35	42	
Yes	35	11	24		12	23	

**Table 2:** Association of iASPP and METTL3 expression in cervical cancer patients.

	Number of cases	METTL3 expression		
		low	high	*P* value
iASPP low	43	26	17	0.002
iASPP high	69	21	48	

**Table 3:** Multivariate survival analysis of the association between prognostic variables and survival in cervical cancer patients.

Variables	5-year RFS			5-year DMFS			5-year PFS			5-year OS		
	HR	95% CI	*P value*	HR	95% CI	*P value*	HR	95% CI	*P value*	HR	95% CI	*P value*
Age (years)	1.376	0.564-3.358	0.483	0.564	0.214-1.487	0.247	0.969	0.475-1.978	0.931	0.698	0.226-2.156	0.532
FIGO staging	5.447	1.949-15.225	0.001	5.608	1.119-28.101	0.036	4.555	1.951-10.635	<0.001	5.462	1.308-22.801	0.02
Tumor size	1.534	0.481-4.887	0.47	1.418	0.523-3.846	0.492	1.781	0.67-4.73	0.247	3.029	0.716-12.823	0.132
Deep cervical stromal invasion	1.066	0.418-2.721	0.893	1.343	0.256-7.044	0.727	1.216	0.571-2.592	0.612	0.467	0.156-1.393	0.172
Lymphovascular space invasion	1.272	0.424-3.811	0.668	1.264	0.421-3.796	0.677	1.684	0.684-4.145	0.256	1.165	0.325-4.183	0.814
Pelvic lymph node metastasis	3.315	1.365-8.05	0.008	2.583	1.109-6.013	0.028	2.408	1.168-4.964	0.017	3.277	1.054-10.19	0.04
Neoadjuvant chemotherapy	1.445	0.247-8.465	0.683	0.437	0.091-2.091	0.3	0.515	0.135-1.962	0.331	0.398	0.039-4.057	0.437
iASPP expression	3.626	1.099-11.965	0.034	4.145	1.527-11.249	0.005	3.505	1.476-8.325	0.004	9.152	1.867-44.869	0.006
METTL3 expression	4.059	1.029-16.019	0.045	19.237	3.973-93.151	0.001	7.197	2.322-22.302	0.001	14.6	2.341-91.05	0.004
